# Effects of β-glucan with vitamin E supplementation on the physiological response, litter performance, blood profiles, immune response, and milk composition of lactating sows

**DOI:** 10.5713/ab.22.0204

**Published:** 2022-09-02

**Authors:** Tae Wook Goh, Jinsu Hong, Hong Jun Kim, Sun Woo Kang, Yoo Yong Kim

**Affiliations:** 1 Department of Agricultural Biotechnology and Research Institute of Agriculture and Life Sciences, Seoul National University, Seoul 08826, Korea; 2 Department of Animal Science, South Dakota State University, Brookings, SD 57006, USA

**Keywords:** β-Glucan, Lactating Sow, Piglet, Piglet Growth Performance, Vitamin E

## Abstract

**Objective:**

This study was conducted to evaluate the effects of β-glucan with vitamin E supplementation on the physiological response, litter performance, blood profiles, immune response, and milk composition of lactating sows.

**Methods:**

A total of 50 multiparous F_1_ sows (Yorkshire×Landrace) with an average body weight (BW) of 233.6±4.30 kg and an average parity of 4.00±0.307 and their litters were used in this experiment. All sows were allotted to one of five treatments, taking into consideration BW, backfat thickness, and parity in a completely randomized design with 10 replicates. The experimental diets included a corn–soybean meal-based basal diet with or without 0.1% or 0.2% β-glucan and 110 IU vitamin E/kg diet.

**Results:**

All treatments added with β-glucan or vitamin E were statistically higher in the average daily feed intake (ADFI) of lactating sows compared to those of the control (Diet, p<0.01). Additionally, the ADFI of lactating sows was significantly higher in the groups supplemented with 0.1% β-glucan compared to 0.2% β-glucan (BG, p<0.01). There was an increasing trend in piglet weight at weaning (BG, p = 0.07), litter weight at the 21st day of lactation (BG, p = 0.07) and litter weight gain (BG, p = 0.08) in groups supplemented with 0.1% β-glucan. The addition of 110 IU vitamin E/kg diet increased vitamin E concentration significantly in lactating sows (VE, p<0.01) and exhibited a trend for higher concentrations of vitamin E (VE, p = 0.09) in piglets. Adding 0.1% β-glucan compared to 0.2% β-glucan induced a decrease in the concentration of tumor necrosis factor-α in lactating sows (BG, p = 0.06) and in piglets (BG, p = 0.09) on the 21st day of lactation. There were no significant differences in the milk composition of sows.

**Conclusion:**

Adding 0.1% β-glucan and 110 IU vitamin E/kg to a lactating sow’s diet was beneficial to the growth performance of piglets by leading to an increase in the feed intake of sows and efficiently supplying vitamin E to both the sows and piglets.

## INTRODUCTION

Much effort has been made to develop feed that meets the increase in litter size and milk production of improved sows. In addition, milk demand by piglets increased as the number of litters increased, but the production of sow’s milk has been insufficient [1]. Therefore, proper nutritional management to improve sow productivity is a major issue in the swine industry. β-Glucan is one of many feed additives used for improving the performance of piglets through the nutritional management of sows and for increasing the milk production and productivity of sows [2].

Oxidative stress can negatively affect the fertility of animals and animal welfare in animals with genetic improvement. Oxidative stress is known to cause fetal growth disorders, infertility, and stillbirth, especially during gestation, and is involved in the occurrence of various complications by damaging deoxyribonucleic acid in the sow’s body and destroying normal cells [3]. Therefore, the defense mechanism of antioxidants such as vitamin E against oxidative stress is important [4].

β-Glucan is a type of functional polysaccharide produced by bacteria, yeast, fungi, or cereals. It has various biological functions, such as improving immunity, preventing disease, and controlling blood glucose levels. According to previous studies, β-glucan is known to improve growth performance when added to swine feed [5]. The addition of β-glucan to swine diets can control the synthesis of the inflammatory cytokines tumor necrosis factor-α (TNF-α) and interleukin-6 (IL-6) and regulate the metabolism of nutrients that negatively affect growth performance [6]. In addition, it can increase phagocytosis and the production of IL-2 and decrease the concentrations of cortisol and TNF-α in the blood [7].

Vitamin E is a fat-soluble substance that exists in eight compound forms (α-, β-, γ- and δ-tocopherols and tocotrienols) that function as antioxidants in plant and animal tissues. α-Tocopherol is known to have the highest physiological activity among isomers of vitamin E in animal bodies [8]. Vitamin E acts as an antioxidant at the cell membrane level, and its deficiency can cause the degeneration of skeletal and cardiac muscle, gastric ulcer formation, and anemia. Vitamin E performs several functions related to reproduction. The amount of maternal vitamin E transferred through the placenta during gestation is very small, and newborn piglets must obtain their daily needs from colostrum or milk. When vitamin E was added to feed of lactating sows, the level of vitamin E in the milk of sows was increased to prevent vitamin E deficiency in piglets and improve the health of sows [9–11].

As described above, there have been many studies de scribing the effects and advantages of β-glucan or vitamin E supplementation. However, there have been no experiments on the effects of supplementing diets with β-glucan and vitamin E together on reproductive performance, litter performance, blood profiles, immune response, or milk composition in lactating sows. Therefore, this study was conducted to evaluate the effects of β-glucan with vitamin E supplementation on the physiological responses, litter performance, blood profiles, immune response, and milk composition of lactating sows.

## MATERIALS AND METHODS

All experimental procedures involving animals were conducted in accordance with the Animal Experimental Guidelines provided by the Seoul National University Institutional Animal Care and Use Committee (SNUIACUC; SNU-200 209-2)

### Experimental animals and housing environment

A total of 50 multiparous F_1_ sows (Yorkshire×Landrace) with an average body weight (BW) of 233.6±4.30 kg and an average parity of 4.00±0.307 and their litters were used in this experiment. All sows were allotted to one of five treatments taking into consideration BW, backfat thickness, and parity in a completely randomized design with 10 replicates. During the lactating period, the animals were raised in a farrowing frame (2.5×1.8 m^2^), and the temperature and ventilation of the farrowing house were automatically controlled by a ventilation fan and an automatic control device. Piglets were cut off the umbilical cord and had their tails cut 3 days postpartum, and male piglets were castrated. Additionally, iron preparations (150 mg/mL) were also injected once.

### Experimental design and diet

The dietary treatments included i) CON, corn–soybean meal (SBM)-based diet; ii) LB, corn-SBM-based diet + 0.1% β-glucan; iii) LBE, corn-SBM-based diet + 0.1% β-glucan + 110 IU vitamin E/kg; iv) HB, corn-SBM-based diet + 0.2% β-glucan; and v) HBE, corn-SBM-based diet + 0.2% β-glucan + 110 IU vitamin E/kg. A corn-SBM-based diet was used as feed in this experiment, and all nutrients in this experimental diet met or exceeded the nutrient requirements of the National Research Council (NRC) [12] on lactating sows. Choline was not added to the premix, and 0.1% choline-chloride was fed in the same way as the experimental feed. In the present study, β-glucan and vitamin E products were provided by E&T company (E&T CO., Ltd, Daejeon, Korea). β-Glucan product consists of cell walls of brewer’s yeast (100% *Saccharomyces cerevisiae*), rich in (1,3)-(1,6)-B-D-glucans and mannans. It is fine solid powder with beige to light brown color. Vitamin E was in the form of vitamin E-acetate. In the case of vitamin E, 68 IU/kg was present in the vitamin premix, and 110 IU/kg of vitamin E was additionally supplemented to the LBE and HBE treatments. All nutrient contents in the feed were formulated equally, and the formula and chemical composition of the experimental diet are presented in Table 1. The crude protein content in the lactating sow feed was 13.43%, the lysine content was 0.96%, the methionine content was 0.29%, the calcium content was 0.90%, and the total phosphorus content was 0.70%.

### Physiological response

The live BW and backfat thickness of sows were measured at 24 hours postpartum and on day 21 of lactation. The BW of the sow was measured by an electric scale (CAS Co. Ltd., Yangju-si, Gyeonggi-do, Korea), and backfat thickness was measured at the P_2_ position (the mean value from both sides of the last rib and 65 mm away from the backbone) by an ultrasound device (Lean Meter, Renco Corp., Minneapolis, MN, USA). Daily feed waste was recorded during lactation to identify the real feed intake of each sow during the experimental period. The weaning-to-estrus interval (WEI) of sows was monitored after weaning as an important parameter for evaluating reproductive performance.

### Litter performance

The BW and average daily gain (ADG) of piglets were measured within 24 hours postpartum and after 21 days of lactation to determine the growth performance of piglets and the lactating ability of sows with initiation of lactation after cross fostering.

### Blood profiles and immune response

Blood samples (n = 5 for each treatment) were collected from the jugular vein of sows using 10 mL disposable syringes at 24 hours postpartum and after 21 days of lactation. Additionally, blood samples (n = 5 for each treatment) were collected from the jugular vein of piglets using 3 mL disposable syringes at 24 hours postpartum and 5 mL disposable syringes at 21 days of lactation. All blood samples were collected in serum tubes (SSTTM II Advance; BD Vacutainer, Becton Dickinson, Plymouth, UK) and centrifuged at 1,957×g and 4°C for 15 min (5810R; Eppendorf, Hamburg, Germany). Subsequently, the supernatant was separated in a microtube (AXYGEN. INC, Union City, CA, USA) and the samples of vitamin E, IL-6, and TNF-α were stored at −20°C, while the samples of selenium and lymphocytes were stored at 4°C for analysis. Each measurement was conducted using the following analysis machines and techniques: Se (Inductively coupled plasma–mass spectrometry [ICP–MS]; Perkin Elmer, Rodgau, Germany), vitamin E (high-performance liquid chromatography [HPLC]; HPLC-UVD; PerkinElmer, Milford, MA, USA), lymphocytes (Flow cytometry, automatic blood analyzer; Sysmex, Hyogo, Japan), TNF-α (Fluorescent, Luminex; Millipore, Austin, TX, USA), and IL-6 (Fluorescent, Luminex; Millipore, USA).

### Milk composition

Colostrum samples (n = 4 for each treatment) were collected from functional mammary glands at 24 hours postpartum, and milk samples (n = 4 for each treatment) were taken at 21 days of lactation. Colostrum and milk were collected from the first and second teats by injecting 10 IU/mL oxytocin (Komi oxytocin inj.; Komipharm International Co. Ltd., Siheung, Gyeonggi, Korea) into the blood vessel of the sows’ ear and stored in 50 mL conical tubes (SPL Life Sciences Co., Ltd., Pocheon-si, Gyeonggi-do, Korea). The collected samples were stored in a freezer (−20°C) until further analysis. Proximate analyses for casein, fat, protein, lactose, total solid and solid not fat (SNF) compositions of milk as well as colostrum were determined using a MilkoScan FT 120 (FOSS, Hillerod, Denmark).

### Statistical analysis

All obtained data were processed by Excel 2010 first, and then analyzed by one-way analysis of variance procedure using Statistical Analysis System 9.4 TS1M7 (SAS Inst. Inc., Cary, NC, USA). Individual sows and their litters were used as the experimental unit in the physiological response, litter performance, blood profiles, immune response, and milk composition. The orthogonal polynomial contrasts were used to determine the effects of diet (β-glucan and vitamin E against the control), β-glucan, vitamin E, as well as the interaction between β-glucan and vitamin E. Data were presented as means and their pooled standard errors. The differences were considered as statistically significant when p<0.05, while 0.05≤p<0.10 was considered to indicate a trend in the data.

## RESULTS AND DISCUSSION

### Physiological response

The effects of β-glucan with vitamin E supplementation in the lactating sow diet on BW, backfat thickness, average daily feed intake (ADFI) and WEI during lactation are shown in Table 2. There were no significant differences in BW and backfat thickness of sows at 24 hours postpartum or on day 21 of lactation. Moreover, WEI was not affected by β-glucan and vitamin E. However, the ADFI of lactating sows were statistically higher in all treatments added with β-glucan or vitamin E than in the control (Diet, p<0.01). Additionally, the ADFI of lactating sows was significantly higher in the treatments with 0.1% β-glucan than in the treatments with 0.2% β-glucan (BG, p<0.01).

Szuba-Trznadel et al [13] conducted an experiment by adding different β-glucan percentages (0%, 0.01%, 0.02%, and 0.03%) in sow feed. β-Glucan had no effect on the BW and backfat thickness of sows between treatments. In addition, no significant differences were found in BW and backfat thickness of sows between treatments according to a different study by Shelton et al [11], which was conducted with the addition of varying concentrations of vitamin E (15, 30, 45, 60 IU D-α-TAc and 44, 66 IU DL-α-TAc). In most previous studies, as in the results of the current experiment, there was no statistically significant difference in BW and backfat thickness of sows when β-glucan and vitamin E were supplemented in the sow feed [10,14,15]. In the present experiment, when β-glucan and vitamin E were added to the feed of lactating sows, the feed intake of lactating sows was significantly increased. Therefore, we concluded that there was no negative effect on BW and backfat thickness, as there was no BW and backfat thickness loss in sows after consuming sufficient nutrients for pig milk production.

In general, it is known that the nutritional and metabolic status of the sows during the lactation period affected their WEI [16]. When the ADFI of lactating sows was low, the postweaning health status of lactating sows became poor, which led to an increase in the WEI [17]. Additionally, it is known that WEI is affected by the lactation period, weight loss during lactation, and the number of piglets [18]. Since there were no significant differences in BW, backfat thickness, or the number of piglets, we concluded that no significant difference was observed even in the WEI.

The feed intake of lactating sows had a strong relationship with changes in weight and backfat thickness. If sufficient nutrients for milk production were not consumed by sows, the nutrients accumulated in the body during gestation were used for milk production, which may negatively affect subsequent reproductive performance [17]. Shen et al [19] conducted an experiment comparing sow feed with 15 g/d topdressing of β-glucan during the lactation period with the control group and reported that there was no significant difference in ADFI during the experimental period. In an experiment by Kim et al [20], no significant difference was found in the ADFI of primiparous and multiparous sows compared to the control when the sows were fed a diet top-dressed with 15 g/d β-glucan.

However, in the current experiment, all treatments with β-glucan supplementation exhibited significantly higher ADFI than the control (p<0.01). The mechanism for the improvement of growth performance after the addition of β-glucan in swine feed is not precisely known [7,21]. However, β-glucan has been shown to improve growth performance by promoting gut health and body immunity in several other animal studies, including mice, chickens, and cattle [22]. In addition, β-glucan is a type of natural immune enhancer that increases the mucosal function of the intestinal wall and improves the gastrointestinal environment. This contributed to the growth of animals such as pigs by aiding the digestion of nutrients in the stomach and improving absorption into the body through the intestinal mucosa [22,23]. Regarding the studies mentioned above, we considered that the ADFI was higher in the experimental groups than the control group because the immunity and health status of the lactating sows improved. In addition, the treatments supplemented with 0.1% β-glucan exhibited significantly higher ADFI than the treatments supplemented with 0.2% β-glucan (BG, p<0.01). Szuba-Trznadel et al [13] conducted an experiment by adding varying levels of β-glucan extracted from yeast to the feed of lactating sows (0%, 0.01%, 0.02%, and 0.03%). The ADFI increased linearly from 0% to 0.02% β-glucan treatments, but decreased at 0.03%. Li et al [6] supplemented the feed of weaning pigs with varying levels of β-glucan (0%, 0.0025%, 0.005%, 0.01%, and 0.02%) and observed that ADFI increased from 0% to 0.005% in β-glucan supplemented groups but decreased in the 0.01% group. As in the results of the previous studies described above, various results have been obtained depending on the level of β-glucan added. Differences in the purity, form, and physical state of β-glucan may have influenced these results [6]. Additionally, it was thought that various amounts of β-glucan can induce or inhibit the expression of inflammatory cytokines, thus promoting the secretion of proinflammatory cytokines and decreasing the secretion of anti-inflammatory cytokines, which may have affected feed intake.

In conclusion, when β-glucan and vitamin E were supple mented in the feed of lactating sows, the addition of β-glucan increased the feed intake of the lactating sows, and the feed intake was the highest when 0.1% β-glucan was added.

### Litter performance

The effects of β-glucan with vitamin E supplementation in the lactating sow diet on litter performance during lactation are shown in Table 3. There were no significant differences in the number of piglets after cross fostering, the number of weaning pigs, litter birth weight, or piglet birth weight. However, there was an increasing trend in piglet weight at weaning (BG, p = 0.07), litter weight at the 21st day of lactation (BG, p = 0.07) and litter weight gain (BG, p = 0.08) in treatments supplemented with 0.1% β-glucan compared to treatments supplemented with 0.2% β-glucan. In addition, piglet weight at weaning was significantly higher in all treatments added with β-glucan or vitamin E than in the control (Diet, p<0.01).

Lactating sows must be supplied with sufficient nutrients to meet the nutritional requirements for body maintenance immediately after farrowing, and an *ad libitum* feeding system was implemented after the feed intake was maximized [18]. Previous studies have reported that *ad libitum* feeding during lactation increased the total feed intake of piglets [23]. In addition, litter weight gain increased in cases of lactating sows with a high feed intake. Lactating sows need sufficient nutrients for milk production. When the feed intake of lactating sows was increased, their milk production eventually improved.

Szuba-Trznadel et al [13] reported that there was no sig nificant difference in the growth performance of piglets even when 0.02% or 0.03% β-glucan was added to lactating and gestating sow diets. Likewise, the addition of varying levels β-glucan to the sow diet (0.5%, 1%, 2%, 0.15%, and 0.3%) had no effect on piglet weight or on the number of weaning pigs [15]. However, the ADG and weaning weight of piglets were improved when 4% β-glucan extracted from yeast was supplemented in lactating and gestating sow diets [24]. In addition, Li et al [6] reported that the ADG of piglets was increased when 0.005% β-glucan was added to a lactating sow diet. According to experiments by Hiss and Sauerwein [25], the ADG and weaning weight of piglets were improved when 0.015% to 0.03% β-glucan was added to the lactating sow diet. It is commonly known that the addition of more than 44 IU of vitamin E to diets does not adversely affect the health status and reproductive performance of sows [9,10]. Shelton et al [11] reported that there was no significant difference in the reproductive performance of sows and the growth performance of piglets, even when vitamin E (15, 30, 45, 60 IU D-α-TAc and 44, 66 IU DL-α-TAc) was added to the gestating and lactating sow diets. Mahan et al [15] reported that the addition of 30 or 60 IU of vitamin E to the sow diet did not show a positive effect on the growth performance of the piglets.

In the present experiment, piglet weight at weaning (BG, p = 0.07), litter weight on the 21st day of lactation (BG, p = 0.07) and litter weight gain (BG, p = 0.08) exhibited an increasing trend when β-glucan was added to the lactating sow diet. This was because pig milk production increased due to the high feed intake of lactating sows, as observed in previous studies. Accordingly, it was hypothesized that the growth performance of the piglets increased when they received sufficient nutrients. Additionally, the effect of adding vitamin E was not observed in the current experiment, which is similar to previous studies by Mahan [15] and Shelton et al [11].

In conclusion, the addition of 0.1% β-glucan in the diets of lactating sows had a positive effect on piglet weight at weaning, litter weight at the 21st day of lactation and litter weight gain.

### Blood profiles

The effects of β-glucan with vitamin E supplementation in the lactating sow diet on blood profiles during lactation are shown in Table 4. There was no significant difference in the concentration of selenium in lactating sows and piglets. However, treatments supplemented with 110 IU vitamin E/kg compared to treatments supplemented with non-additional vitamin E at 21 days of lactation showed a significantly higher vitamin E concentration (VE, p<0.01) in lactating sows and an increasing trend in vitamin E concentration (VE, p = 0.09) in piglets. In addition, vitamin E concentration at 21 days of lactation in lactating sows showed an increasing trend in all treatments added with β-glucan or vitamin E than in the control (Diet, p = 0.06).

When vitamin E was supplemented at different concen trations (44 IU and 66 IU) starting on 70 days of gestation and ending prior to weaning in a study by Shelton et al [11], the concentration of α-tocopherol in the plasma of sows increased linearly at 100 days of gestation, within 24 hours of parturition and during weaning. In addition, Mahan [10] also showed that the concentration of α-tocopherol in the serum of lactating sows and piglets was significantly increased as vitamin E was added at different levels (22 IU, 44 IU, and 66 IU) in the lactating sow diet. In a study by Umesiobi [26], the concentration of α-tocopherol significantly increased as the level of vitamin E addition increased (0 IU, 40 IU, and 70 IU) in the feed of 1st parity sows and 2nd parity sows. Wang et al [27] conducted an experiment comparing a control group in which 44 IU/kg vitamin E was added to a basal diet and a treatment group in which a total of 250 IU/kg of vitamin E was added from the 107th day of gestation until the piglets were weaned. Their results showed that the concentration of α-tocopherol in the blood of lactating sows and piglets was significantly higher in the treatment group with a total of 250 IU/kg vitamin E than in the control (p<0.01).

Due to limited movement of nutrients from the mother to the fetus through the placenta during gestation, piglets are deficient in vitamin E immediately after birth, so α-tocopherol must be supplied through the colostrum and pig milk of sows [9]. Insufficient intake of milk by piglets may lead to a decrease in serum vitamin E levels during weaning, which may increase oxidative status and susceptibility to disease.

In the current experiment, we found that the concentra tion of α-tocopherol in the blood of lactating sows and piglets was increased when vitamin E was added to the lactating sow diet. The increase in the concentration of vitamin E in the blood was consistent with the results of previous studies by Mahan et al [10], Shelton et al [11], Umesiobi et al [26], and Wang et al [27]. This showed that when vitamin E was additionally supplied to lactating sows, the effect was that the concentration of vitamin E in the blood increased in the sows. The concentration of vitamin E in the piglets was also increased, as in previous studies by Mahan et al [15] and Wang et al [27]. This showed the same result that the concentration of vitamin E in the blood of lactating sows increased as the concentration of vitamin E added to the lactating sow diet increased. This suggests that vitamin E was also well delivered to piglets through pig milk. Additionally, it can be expected that this will have a positive effect on the reduction of oxidative stress and disease susceptibility of piglets. In addition, it can be reasoned that this helps to improve immunity not only in lactating sows but also in piglets [27].

In conclusion, the addition of 110 IU vitamin E/kg to lac tating sow feed had a positive effect on the concentration of vitamin E in the blood of lactating sows and piglets.

### Immune response

The effects of β-glucan with vitamin E supplementation in the lactating sow diet on immune response during lactation are shown in Table 5. The results show that there was no significant difference in the concentration of lymphocytes and IL-6 in lactating sows and piglets. However, compared to treatments supplemented with 0.2% β-glucan, treatments supplemented with 0.1% β-glucan exhibited a decrease in the concentration of TNF-α (BG, p = 0.06) in lactating sows and piglets (BG, p = 0.09) on the 21st day of lactation.

In general, piglet immunity is derived from the absorp tion of immune proteins in the colostrum and from immune proteins synthesized in the body after 35 days. Lymphocytes are part of the adaptive immune system and account for 20% to 40% of white blood cells. TNF-α and IL-6 secretion indicates excessive inflammatory responses, including the expression of inflammatory cytokines. TNF-α is a proinflammatory cytokine that activates adaptive immunity against viral infections. TNF-α blood levels have been used as an indicator of inflammation in humans and animals. Generally, proinflammatory cytokines such as TNF-α negatively affect animal growth and well-being when they are increased. However, many cytokines play a very important role in enhancing the innate immune response and directing adaptive immunity against responses dominated by Th1 or Th2 cells. β-Glucan is well known as an important stimulator of the cellular branch of the immune response and improves biological and immunological status [21]. Additionally, it partially inhibits or reduces the increase in TNF-α production upon endotoxin and lipopolysaccharide (LPS) challenge [6,28].

TNF-α blood levels (p <0.05) were decreased on the 7th day when weaning pigs were fed a 0.05% yeast cell wall product containing β-glucan [29]. This was explained by the increased antibody production observed after immune system stimulation as a result of binding with the β-glucan receptor and by triggering a cytokine cascade and improving macrophage function. The ileal expression of TNF-α mRNA was higher in weaning pigs fed 10 g/d of seaweed extract, mainly containing laminarians composed of β-glucan, than in control weaning pigs (p<0.01) [30]. Li et al [6] conducted a 2×2 factorial experiment in weaning pigs with and without LPS inoculation and with or without β-glucan addition (0% or 0.005%). The results show that TNF-α levels (p<0.05) were lower in weaning pigs supplemented with 0.005% β-glucan at 3, 9, 18, and 24 hours, regardless of LPS inoculation. In addition, TNF-α levels were increased by LPS at all time points except at 0 hour (p<0.05). When weaning pigs inoculated with LPS were fed a diet supplemented with β-glucan extracted from 0.005% baker’s yeast (*Saccharomyces cerevisiae*), TNF-α levels in the blood were lower at 3 hours (p<0.05) and 4.5 hours after injection than they were in control pigs [6]. As a result of an experiment on the effect of adding vitamin C and β-glucan to weaning pigs after endotoxin challenge, the spleen of weaning pigs fed 2.5% β-glucan expressed a higher relative abundance of TNF-α mRNA compared to the control pigs (p<0.01) [28].

In pigs, an increase in inflammatory cytokines such as TNF-α resulted in the distribution of nutrients to the immune system rather than growth, leading to decreased net muscle accumulation and growth [30]. This indicated that since the TNF-α concentration in the treatments supplemented with 0.1% β-glucan showed a decreasing trend, more nutrients were distributed for animal growth. This is evident from the significantly higher ADFI of lactating sows in the treatments supplemented with 0.1% β-glucan compared to 0.2% β-glucan (BG, p<0.01) and from the increase in piglet weight at weaning (BG, p = 0.07), litter weight at the 21st day of lactation (BG, p = 0.07) and litter weight gain (BG, p = 0.08).

In conclusion, the TNF-α concentration was thought to be decreased as the immune status of the sows and piglets was improved when 0.1% β-glucan was added to the lactating sow diet.

### Milk composition

The effects of β-glucan with vitamin E supplementation in the lactating sow diet on milk composition during lactation are shown in Table 6. The results show that there were no significant differences in casein, protein, fat, total solids, SNF, or lactose in the milk of lactating sows.

Various factors, such as breed, sow health, feeding pro gram, nutritional level, and environmental conditions, affect sow milk composition and production. Milk composition has been shown to affect the growth of piglets [19]. Nutrient requirements in lactating sows are significantly higher than those during the gestation period. Lactating sows were generally fed *ad libitum* for optimal milk production, maintenance and maternal recovery.

In the present experiment, there was no significant differ ence in the milk composition of sows between the treatments after the addition of β-glucan and vitamin E. Since the nutrients accumulating in the body of the sows were preferentially transferred to the pig’s milk, the effect on the feed intake during the lactation period was small. Thus, we concluded that the components of the sow milk were not affected.

As shown here, Shen et al [19] reported that feeding *Saccharomyces cerevisiae* fermentation product to sows during gestation and lactation did not affect the composition of the colostrum and milk. In the study by Chen et al [31], colostrum and milk components were not affected when *Saccharomyces cerevisiae* fermentation product was added in a subtropical climate.

In conclusion, the addition of β-glucan and vitamin E to the feed of lactating sows did not have a positive effect on the components of sow milk.

## CONCLUSION

The addition of 0.1% β-glucan improved the growth performance of the piglets by providing them with sufficient nutrients. This was because the production of pig milk improved as the feed intake of the sows increased. In addition, treatments with 0.1% β-glucan induced a lower TNF-α concentration than the treatments with 0.2% β-glucan. This meant that the inflammatory response in the lactating sows and piglets was reduced and the health status was improved, which was thought to be the result of the improvement in the growth performance of the piglets. Furthermore, the addition of 110 IU vitamin E/kg increased the concentration of α-tocopherol in the blood of the sows and piglets, indicating that addition of vitamin E was better delivered to the sows and piglets. However, as the feed intake of sows increased, a more positive effect on the growth performance of the piglets was found when β-glucan was added at 0.1% rather than 0.2%. Therefore, adding 0.1% β-glucan and 110 IU vitamin E/kg to the feed of the sows was beneficial to the growth performance of the piglets by increasing the feed intake of the sows and efficiently supplying vitamin E to the sows and piglets.

## Figures and Tables

**Table 1:** Formula and chemical compositions of the experimental diets during the lactation phase

Items	Treatment^1)^				

	CON	LB	LBE	HB	HBE
Ingredient (%)					
Corn	66.19	66.12	66.10	66.09	66.04
Soybean meal-46%	15.32	15.29	15.28	15.22	15.23
Wheat	5.00	5.00	5.00	5.00	5.00
Wheat bran	5.00	5.00	5.00	5.00	5.00
Vitamin E^2)^	0.00	0.00	0.02	0.00	0.02
β-Glucan^2)^	0.00	0.10	0.10	0.20	0.20
Tallow	3.58	3.58	3.59	3.58	3.60
L-lysine HCl (78%)	0.43	0.43	0.43	0.43	0.43
DL-methionine (99%)	0.07	0.07	0.07	0.07	0.07
L-threonine (99%)	0.11	0.11	0.11	0.11	0.11
Tryptophan (10%)	0.31	0.31	0.31	0.31	0.31
MDCP	1.86	1.86	1.86	1.86	1.86
Limestone	1.33	1.33	1.33	1.33	1.33
Vitamin. Mix^3)^	0.10	0.10	0.10	0.10	0.10
Mineral. Mix^4)^	0.10	0.10	0.10	0.10	0.10
Choline chloride-50%	0.10	0.10	0.10	0.10	0.10
Salt	0.50	0.50	0.50	0.50	0.50
Sum	100.00	100.00	100.00	100.00	100.00
Chemical composition^5)^					
ME (kcal/kg)	3,300.00	3,300.00	3,300.00	3,300.00	3,300.00
Crude protein (%)	13.43	13.43	13.43	13.43	13.43
Lysine (%)	0.96	0.96	0.96	0.96	0.96
Methionine (%)	0.29	0.29	0.29	0.29	0.29
Cysteine (%)	0.27	0.27	0.27	0.27	0.27
Threonine (%)	0.61	0.61	0.61	0.61	0.61
Tryptophan (%)	0.17	0.17	0.17	0.17	0.17
Calcium (%)	0.90	0.90	0.90	0.90	0.90
Total phosphorus (%)	0.70	0.70	0.70	0.70	0.70

MDCP, mono-dicalcium phosphate; ME, metabolizable energy; SBM, soybean meal

1) CON, corn-SBM based diet; LB, corn-SBM based diet+0.1% β-glucan; LBE, corn-SBM based diet+0.1% β-glucan+110 IU vitamin E/kg; HB, corn-SBM based diet+0.2% β-glucan; HBE, corn-SBM based diet+0.2% β-glucan+110 IU vitamin E/kg.

2) β-glucan and vitamin E products were provided by E&T company (E&T Co., Ltd, Daejeon, Korea).

3) Provided the following quantities of vitamins per kg of complete diet: Vitamin A, 12,000 IU; Vitamin D_3_, 1,200 IU; Vitamin E, 68 IU; Vitamin K_3_, 5 mg; Thiamin, 2.6 mg; Rivoflavin, 8 mg; Calcium pantothenic acid, 26 mg; Niacin, 60 mg; D–Biotin, 0.5 mg; Vitamin B_6_, 6 mg; Vitamin B_12_, 0.025 mg.

4) Provided the following quantities of minerals per kg of complete diet: Fe, 120 mg; Cu, 6 mg; Mn, 60 mg; I, 0.75 mg; Se, 0.3 mg; Zn, 46 mg.

5) Calculated value.

**Table 2:** Effects of β-glucan with vitamin E supplementation on the physiological response in lactating sows

Items	Treatment^1)^					SEM^2)^	p-value			
	
	CON	LB	LBE	HB	HBE		Diet	BG	VE	BG×VE
Body weight (kg)										
24 hours postpartum	231.90	231.13	236.39	235.76	231.14	4.302	0.87	0.97	0.97	0.62
21st day of lactation	232.88	237.81	234.54	232.68	228.21	4.857	0.97	0.61	0.73	0.95
Changes (0 to 21 d)	0.98	6.68	−1.85	−3.08	−2.93	2.612	0.84	0.36	0.49	0.47
Backfat thickness (mm)										
24 hours postpartum	22.00	22.95	23.00	23.45	22.45	0.693	0.59	0.98	0.77	0.75
21st day of lactation	20.65	20.60	21.65	21.85	20.95	0.697	0.73	0.86	0.96	0.55
Changes (0 to 21 d)	−1.35	−2.35	−1.35	−1.60	−1.50	0.358	0.70	0.71	0.51	0.59
ADFI (kg/d)	5.25	6.59	6.14	6.29	6.01	0.112	<0.01	<0.01	0.17	0.25
WEI (d)	3.41	3.91	3.72	3.66	3.85	0.126	0.30	0.84	0.99	0.51

BG, β-glucan; VE, vitamin E; ADFI, average daily feed intake; WEI, weaning-to-estrus interval; SBM, soybean meal.

1) CON, corn-SBM based diet; LB, corn-SBM based diet+0.1% β-glucan; LBE, corn-SBM based diet+0.1% β-glucan+110 IU vitamin E/kg; HB, corn-SBM based diet+0.2% β-glucan; HBE, corn-SBM based diet+0.2% β-glucan+110 IU vitamin E/kg.

2) Standard error of the mean.

**Table 3:** Effects of β-glucan with vitamin E supplementation on litter performance

Items	Treatment^1)^					SEM^2)^	p-value			
	
	CON	LB	LBE	HB	HBE		Diet	BG	VE	BG×VE
No. of sows	10.00	10.00	10.00	10.00	10.00	-	-	-	-	-
No. of piglets										
Initial	11.30	11.60	11.60	12.00	11.90	0.310	0.97	0.34	0.13	0.13
21st day of lactation	10.00	10.40	10.50	10.60	10.30	0.251	0.75	0.38	0.29	0.22
Litter weight (kg)										
Initial	14.90	17.01	15.91	16.55	15.94	0.443	0.20	0.82	0.40	0.81
21st day of lactation	56.34	66.42	64.53	65.94	61.90	1.765	0.11	0.07	0.13	0.28
Litter weight gain	41.43	49.41	48.62	49.39	45.96	1.638	0.27	0.08	0.11	0.22
Piglet weight (kg)										
Initial	1.32	1.47	1.37	1.38	1.43	0.031	0.25	0.89	0.78	0.37
21st day of lactation	5.70	6.33	6.45	6.24	6.01	0.099	<0.01	0.07	0.18	0.10
Piglet weight gain	4.37	4.87	4.79	4.85	4.57	0.862	0.62	0.64	0.77	0.89

BG, β-glucan; VE, vitamin E; SBM, soybean meal.

1) CON, corn-SBM based diet; LB, corn-SBM based diet+0.1% β-glucan; LBE, corn-SBM based diet+0.1% β-glucan+110 IU vitamin E/kg; HB, corn-SBM based diet+0.2% β-glucan; HBE, corn-SBM based diet+0.2% β-glucan+110 IU vitamin E/kg.

2) Standard error of the mean.

**Table 4:** Effects of β-glucan with vitamin E supplementation on blood profiles in lactating sows and piglets

Items	Treatment^1)^					SEM^2)^	p-value			
	
	CON	LB	LBE	HB	HBE		Diet	BG	VE	BG×VE
Sow										
Selenium (μg/L)										
Initial	------------------------------- 154.2 -------------------------------					-	-	-	-	-
21st day of lactation	208.60	202.00	195.50	208.50	216.00	4.036	0.76	0.17	0.96	0.47
Vitamin E (Tocopherol, μmol/L)										
Initial	--------------------------------- 3.5 ---------------------------------					-	-	-	-	-
21st day of lactation	7.05	7.50	10.78	7.88	9.50	0.399	0.06	0.60	<0.01	0.32
Piglet										
Selenium (μg/L)										
Initial	--------------------------------- 67.2 ---------------------------------					-	-	-	-	-
21st day of lactation	90.20	108.40	85.67	88.75	90.80	4.461	0.77	0.50	0.34	0.26
Vitamin E (Tocopherol, μmol/L)										
Initial	--------------------------------- 9.4 ---------------------------------					-	-	-	-	-
21st day of lactation	8.60	9.70	19.45	15.15	15.93	1.287	0.53	0.64	0.09	0.10

BG, β-glucan; VE, vitamin E; SBM, soybean meal.

1) CON, corn-SBM based diet; LB, corn-SBM based diet+0.1% β-glucan; LBE, corn-SBM based diet+0.1% β-glucan+110 IU vitamin E/kg; HB, corn-SBM based diet+0.2% β-glucan; HBE: corn-SBM based diet+0.2% β-glucan+110 IU vitamin E/kg.

2) Standard error of the mean.

**Table 5:** Effects of β-glucan with vitamin E supplementation on the immune response in lactating sows and piglets

Items	Treatment^1)^					SEM^2)^	p-value			
	
	CON	LB	LBE	HB	HBE		Diet	BG	VE	BG×VE
Sow										
Lymphocyte (%)										
Initial	------------------------------------ 30.2 ------------------------------------					-	-	-	-	-
21st day of lactation	42.50	38.43	42.38	40.67	42.30	1.233	0.64	0.73	0.38	0.71
TNF-α (ng/mL)										
Initial	------------------------------------ 1.06 ------------------------------------					-	-	-	-	-
21st day of lactation	1.45	2.12	2.20	2.33	2.24	0.124	0.89	0.06	0.89	0.17
IL-6 (ng/mL)										
Initial	------------------------------------ 2.42 ------------------------------------					-	-	-	-	-
21st day of lactation	3.61	3.58	3.23	4.41	1.87	0.483	0.78	0.82	0.23	0.36
Piglet										
Lymphocyte (%)										
Initial	------------------------------------ 38.1 ------------------------------------					-	-	-	-	-
21st day of lactation	71.70	67.78	51.11	74.87	78.37	4.709	0.76	0.13	0.55	0.36
TNF-α (ng/mL)										
Initial	------------------------------------ 1.35 ------------------------------------					-	-	-	-	-
21st day of lactation	0.45	0.32	1.39	1.67	1.60	0.230	0.12	0.09	0.26	0.21
IL-6 (ng/mL)										
Initial	------------------------------------ 1.74 ------------------------------------					-	-	-	-	-
21st day of lactation	0.83	1.07	0.78	1.60	1.35	0.215	0.70	0.67	0.12	0.74

BG, β-glucan; VE, vitamin E; SBM, soybean meal; TNF-α, tumor necrosis factor-α; IL, interleukin.

1) CON, corn-SBM based diet; LB, corn-SBM based diet+0.1% β-glucan; LBE, corn-SBM based diet+0.1% β-glucan+110 IU vitamin E/kg; HB, corn-SBM based diet+0.2% β-glucan; HBE, corn-SBM based diet+0.2% β-glucan+110 IU vitamin E/kg.

2) Standard error of the mean.

**Table 6:** Effects of β-glucan with vitamin E supplementation on milk composition in lactating sows

Items	Treatment^1)^					SEM^2)^	p-value			
	
	CON	LB	LBE	HB	HBE		Diet	BG	VE	BG×VE
Casein										
24 h postpartum	------------------------------------ 5.15 ------------------------------------					-	-	-	-	-
21st day of lactation	3.65	3.62	3.63	3.70	3.70	0.044	0.91	0.50	0.99	0.94
Protein										
24 h postpartum	------------------------------------ 7.28 ------------------------------------					-	-	-	-	-
21st day of lactation	4.46	4.39	4.43	4.50	4.59	0.062	0.91	0.38	0.67	0.88
Fat										
24 h postpartum	------------------------------------ 7.80 ------------------------------------					-	-	-	-	-
21st day of lactation	5.73	6.60	7.11	6.69	6.61	0.232	0.10	0.69	0.68	0.58
Total solid										
24 h postpartum	------------------------------------ 21.47 ------------------------------------					-	-	-	-	-
21st day of lactation	17.46	18.31	18.69	18.41	18.35	0.247	0.14	0.83	0.79	0.70
SNF										
24 h postpartum	------------------------------------ 12.50 ------------------------------------					-	-	-	-	-
21st day of lactation	11.19	11.01	10.84	11.06	11.08	0.057	0.19	0.27	0.57	0.47
Lactose										
24 h postpartum	------------------------------------ 4.38 ------------------------------------					-	-	-	-	-
21st day of lactation	5.89	5.79	5.66	5.78	5.69	0.041	0.14	0.89	0.25	0.85

BG, β-glucan; VE, vitamin E; SNF, solid not fat; SBM, soybean meal.

1) CON, corn-SBM based diet; LB, corn-SBM based diet+0.1% β-glucan; LBE, corn-SBM based diet+0.1% β-glucan+110 IU vitamin E/kg; HB, corn-SBM based diet+0.2% β-glucan; HBE, corn-SBM based diet+0.2% β-glucan+110 IU vitamin E/kg.

2) Standard error of the mean.
